# An updated meta-analysis of 37 case-control studies on the association between *NFKB1* −94ins/del ATTG promoter polymorphism and cancer susceptibility

**DOI:** 10.18632/oncotarget.10808

**Published:** 2016-07-24

**Authors:** Yi-Qiao Luo, Duan Wang, Teng Gong, Jiang Zhu

**Affiliations:** ^1^ Department of Thoracic Oncology, West China Hospital/West China School of Medicine, Sichuan University, Chengdu, China; ^2^ Department of Orthopaedics, West China School of Medicine, West China Hospital, Sichuan University, Chengdu, China; ^3^ Sichuan Mianyang 404 Hospital, Mianyang, China; ^4^ Department of Thoracic Oncology, West China Hospital, Sichuan University, Chengdu, China

**Keywords:** NFKB1, polymorphism, cancer, meta-analysis

## Abstract

As a cell survival signal, nuclear factor-kappa B (*NFKB*) is associated with the pathogenesis of numerous malignancies. According to several studies, *NFKB1* −94ins/del ATTG promoter polymorphism is associated with the risk of different malignancies, but the results were not consistent. Therefore, we performed an updated meta-analysis based on 37 case-control studies from 33 articles (16,271 cases and 22,781 controls) to clarify the relationship. The odds ratio (OR) and 95% confidence interval (CI) were used to determine the strength of the association. We found that the *NFKB1* −94ins/del ATTG promoter polymorphism was significantly associated with increased susceptibility to cancer in the recessive (II *vs*. ID+DD, OR = 1.140, 95% CI = 1.029–1.263, *p* =0.012), homozygote (II *vs*. DD, OR = 1.259, 95% CI = 1.068–1.485, *p* =0.006), and allele (I *vs*. D, OR = 1.109, 95% CI = 1.025–1.199, *p* =0.010) genetic models. The subgroup analysis for ethnicity found that the *NFKB1* −94ins/del ATTG promoter polymorphism was significantly associated with an increased susceptibility to cancer in Asians and with a decreased susceptibility in Caucasians. The stratified analyses revealed significant associations between the polymorphism and increased susceptibility to ovarian cancer, oral squamous cell carcinoma, and nasopharyngeal carcinoma.

## INTRODUCTION

Cancer is the result of complex interactions between inherited and environmental factors, which threatens people worldwide due to high morbidity and mortality [1]. Although the aetiology of this disease remains unclear, genetic susceptibility is one known explanation for the inter-individual variation in cancer risk [2]. Many researchers have been studying the aetiology of oncogenesis, and have identified the relationship between genetic polymorphism and cancer risk, especially for the *NFKB1* −94ins/del ATTG promoter polymorphism.

*NFKB* is responsible for regulating the expression of many genes for immune response, cell adhesion, differentiation, proliferation, angiogenesis and apoptosis [3]. *NFKB* was first identified by Sen and Baltimore in 1986 [4]. As a transcription factor, *NFKB* binds to a 10 bp DNA element in kappa immunoglobulin light-chain enhancer in B cells [5]. The *NFKB* family consists of p50/p105, p65/Rel A, c-Rel, Rel B, and p52/p100. Among them, the major form of *NFKB* is a heterodimer of the p50/p105 and p65/Rel A subunits that are encoded by the *NFKB1* and *NFKB2* genes, respectively [49]. The human *NFKB1* gene, located on chromosome 4q24, encodes a 50 kDa DNA-binding protein that can act as a master regulator of inflammation and cancer development [6,7].

A common insertion/deletion polymorphism in the promoter region of the *NFKB1* gene elicits a regulatory effect on the *NFKB1* gene [8] and an increasing number of studies have assessed the association between the *NFKB1* −94ins/del ATTG promoter polymorphism and cancer risk [9–11]. However, some researchers could not replicate this association. Previous meta-analysis [45–48] focused on the relationship between the *NFKB1* −94ins/del ATTG promoter polymorphism and cancer, but the results were inconsistent. Since then, several other studies [36–44] performed on large case and control groups have assessed the relationships between the *NFKB1* −94ins/del ATTG promoter polymorphism and susceptibility to a variety of cancers. Therefore, to better understand the precise relationships, we performed a comprehensive updated meta-analysis with increased statistical power.

## RESULTS

### Characteristics of eligible studies

**Figure 1:**
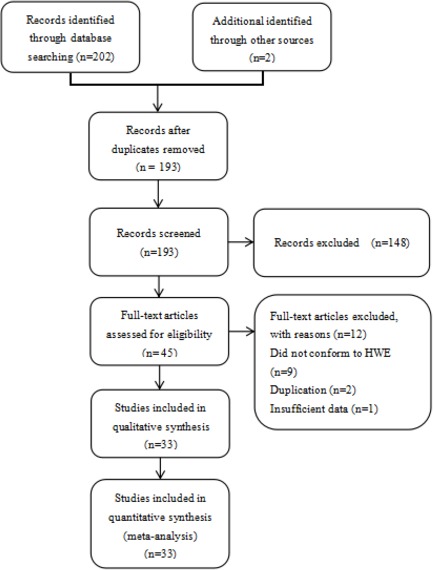
Flow chart of the process for study identification and selection

Our electronic database search resulted in 202 articles and 2 articles were available manually, we scanned all of the abstracts, and there were 45 articles that conformed to the inclusion criteria, we excluded 9 articles [52–60] that did not conform to HWE, 2 studies [61, 62] were excluded as they were duplications of previous publications and 1 study [63] did not have completely extractable data. Thus, we included 33 independent records [14–44, 50–51]. Riemann et al [15] was treated as three independent case groups because three cancer types were studied along with a control sample. Li et al [39] conducted their research in three types of urinary cancer (renal cancer, bladder cancer and prostate cancer), so we treated the data as three separate comparisons. Finally, a total of 37 separate studies involving 16,271 cases and 22,781 controls were available for our updated meta-analysis. Figure 1 describes the process for the study. Characteristics of the eligible studies are summarized in Table 1. Among them, 26 studies were performed in Asian populations and 11 studies in Caucasian populations. In total, this meta-analysis included 5 studies on colorectal cancer studies, 4 on bladder cancer studies, 4 on ovarian cancer studies, 4 on prostate cancer studies, 3 on hepatocellular carcinoma studies, 3 on nasopharyngeal carcinoma studies, 2 on gastric cancer studies, 2 on oral squamous cell carcinoma studies, 2 on non-small cell lung cancer studies, 2 on renal cell cancer studies and 5 on other cancers. All cases were clinically pathologically confirmed.

**Table 1:** Characteristics of studies included in the meta-analysis

Author	Year	Ethnicity	Country	Cases	Control	Method	Cancer type	Case			Control			HWE
								II	ID	DD	II	ID	DD	
Lin	2006	Asian	China	212	201	PCR	OSCC	59	103	50	43	100	58	0.993
Riemann	2006	Caucasian	Germany	139	307	PCR-RFLP	CRC	54	58	27	118	141	48	0.586
Riemann	2006	Caucasian	Germany	72	307	PCR-RFLP	B cell CLL	18	41	13	118	141	48	0.586
Riemann	2006	Caucasian	Germany	140	307	PCR-RFLP	RCC	47	76	17	118	141	48	0.586
Riemann	2007	Caucasian	Germany	242	307	PCR-RFLP	BC	88	124	30	118	141	48	0.586
Lo	2009	Asian	China	182	116	PCR	GC	62	89	31	20	62	34	0.361
He	2009	Asian	China	202	404	PCR-RFLP	HCC	83	84	35	97	183	124	0.07
Zhang	2009	Asian	China	117	143	PCR-PAGE	PC	46	57	14	44	68	31	0.624
Zhou	2009	Asian	China	163	203	PCR-RFLP	NPC	74	67	22	71	90	42	0.177
Zhou	2010	Asian	China	233	365	PCR-PAGE	CSCC	108	105	20	135	166	64	0.297
Andersen	2010	Caucasian	Denmark	378	756	TaqMan	CRC	121	195	62	307	347	102	0.801
Tang	2010	Asian	China	207	228	PCR-PAGE	BC	89	92	26	74	108	46	0.565
Song	2011	Asian	China	1001	1005	PCR-RFLP	CRC	363	500	138	297	522	186	0.102
Fan	2011	Asian	China	179	223	PCR-CE	OC	78	84	17	76	103	44	0.396
Vangsted	2012	Caucasian	Denmark	348	1700	Taqman	MM	110	163	55	665	778	253	0.303
Ungerback	2012	Caucasian	Sweden	344	622	TaqMan	CRC	114	187	43	256	270	96	0.079
Liu	2012	Asian	China	906	906	PCR	NPC	269	467	170	280	433	193	0.289
Lin	2012	Asian	China	462	520	TaqMan	OSCC	116	246	100	81	271	168	0.099
Kopp	2013	Caucasian	Denmark	334	334	TaqMan	PC	128	152	54	109	161	64	0.741
Huo	2013	Asian	China	187	221	PCR	OC	83	82	22	71	103	47	0.399
Cheng	2013	Asian	China	135	520	RT-PCR	HCC	42	64	29	81	271	168	0.099
Li	2013	Asian	China	609	640	TaqMan	BC	189	269	151	223	324	93	0.156
Oltulu	2014	Caucasian	Turkey	95	99	PCR-RFLP	NSCLC	35	44	16	46	47	6	0.18
Hua	2014	Asian	China	401	433	HapMap	GC	92	182	127	120	230	83	0.144
Zhang	2014	Asian	China	624	1606	PCR	HCC	205	312	107	542	790	274	0.63
Liu	2015	Asian	China	1590	1979	HapMap	NPC	552	769	269	610	950	419	0.169
Wang	2015	Asian	China	421	425	PCR-RFLP	NSCLC	113	219	89	89	205	131	0.595
Lu	2015	Asian	China	687	687	PCR-RFLP	OC	115	351	221	95	339	253	0.271
Kopp	2015	Caucasian	Denmark	915	1719	KASP	CRC	320	449	146	679	787	253	0.311
Chen	2015	Asian	China	410	442	PCR	OC	120	195	95	85	235	122	0.136
Li	2015	Asian	China	730	780	TaqMan	BC	227	316	187	261	395	124	0.208
Li	2015	Asian	China	1216	1588	TaqMan	RCC	451	577	188	582	781	225	0.152
Li	2015	Asian	China	820	945	TaqMan	PC	299	377	144	347	462	136	0.371
Wang	2015	Asian	China	352	459	PCR	PTC	106	186	60	171	209	79	0.273
Li	2015	Asian	China	220	222	PCR-RFLP	Osteosarcoma	60	114	46	50	106	66	0.55
Han	2015	Asian	China	936	936	PCR-RFLP	PC	63	339	534	38	331	567	0.23
Rybka	2016	Caucasian	Poland	62	126	PCR	AML	25	30	7	43	69	14	0.079

PTC papillary thyroid carcinoma, CRC colorectal cancer, BC, bladder cancer, OC ovarian cancer, PC prostate cancer, HCC hepatocellular carcinoma, GC gastric cancer, OSCC oral squamous cell carcinoma, NSCLC none small cell lung cancer, NPC nasopharyngeal carcinoma, RCC renal cell carcinoma, MM multiple myeloma, AML acute myeloid leukaemia

### Meta-analysis of the overall population

**Figure 2:**
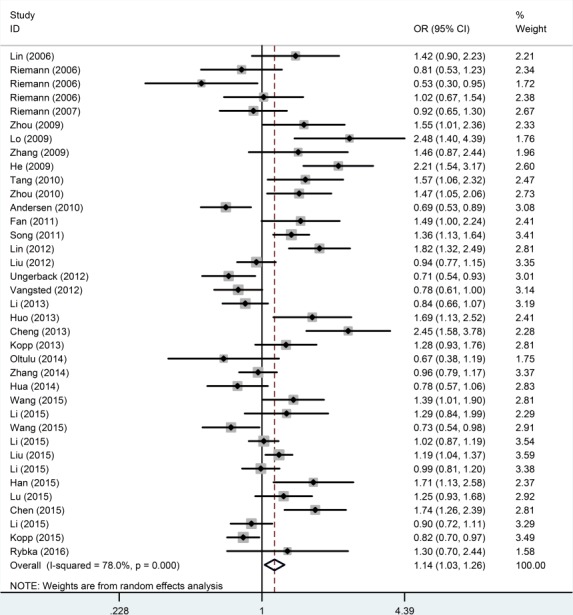
Forest plots of ORs with 95% CI for the *NFKB1* −94ins/del ATTG promoter polymorphism and risk of cancer in the overall population (II *vs*. ID + DD)

The main meta-analysis results of the association between the *NFKB1* −94ins/del ATTG promoter polymorphism and cancer risk are shown in Table 2. All *P* values displayed obvious heterogeneity between the selected research studies under all five genetic models of the updated meta-analysis. Thus, the random-effect model was used. We found that the *NFKB1* −94ins/del ATTG promoter polymorphism was significantly increased cancer risk in homozygote (II *vs*. DD, OR = 1.259, 95% CI = 1.068-1.485), recessive (II *vs*. ID+DD, OR = 1.140, 95% CI = 1.029-1.263) and allele (I *vs*. D, OR = 1.109, 95% CI = 1.025-1.199) genetic models. However, the association was not found in II+ID *vs*. DD (OR = 1.139, 95% CI = 0.994-1.305) and ID *vs*. DD (OR = 1.118, 95% CI = 0.997-1.253). (Figure 2).

### Subgroup analyses

**Figure 3:**
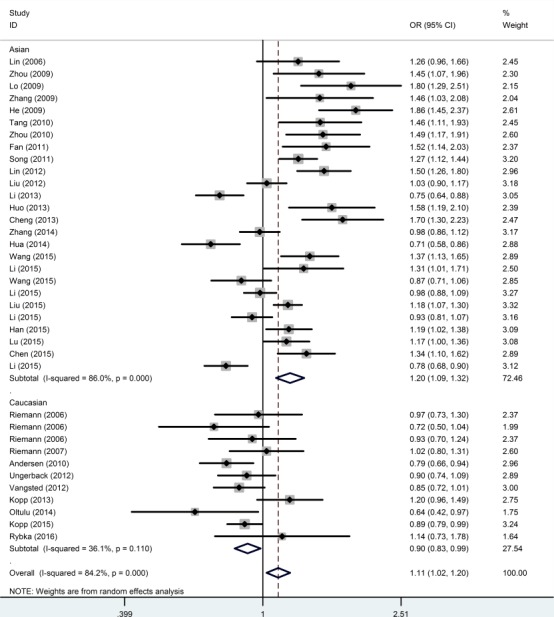
Forest plots of ORs with 95% CI for the *NFKB1* −94ins/del ATTG promoter polymorphism and risk of cancer in ethnicity (I *vs*. D)

The subgroup analysis for ethnicity revealed significant increases in susceptibility for cancer risk in the four models among Asians (II+ID *vs*. DD, OR = 1.223, 95% CI = 1.031-1.451; II *vs*. ID+DD, OR = 1.280, 95% CI = 1.142-1.435; II *vs*. DD, OR = 1.463, 95% CI = 1.196-1.788; I *vs*. D, OR = 1.199, 95% CI = 1.092-1.317) and decreases in susceptibility in three models among Caucasians (II *vs*. ID+DD, OR = 0.824, 95% CI = 0.752-0.903; II *vs*. DD, OR = 0.855, 95% CI = 0.748-0.979; I *vs*. D, OR = 0.899, 95% CI = 0.844-0.958). (Figure 3, Table 2). The stratified analyses revealed a significant association between the polymorphism and ovarian cancer (II+ID *vs*. DD, OR = 1.481, 95% CI = 1.128-1.943; II *vs*. ID+DD, OR = 1.503, 95% CI = 1.265-1.786; II *vs*. DD, OR = 1.761, 95% CI = 1.420-2.184; ID *vs*. DD, OR = 1.246, 95% CI = 1.048-1.482; I *vs*. D, OR = 1.308, 95% CI = 1.181-1.449), oral squamous cell carcinoma (II+ID *vs*. DD, OR = 1.593, 95% CI = 1.253-2.026; II *vs*. ID+DD, OR = 1.674, 95% CI = 1.292-2.169; II *vs*. DD, OR = 2.104, 95% CI = 1.545-2.867; ID *vs*. DD, OR = 1.420, 95% CI = 1.102-1.829; I *vs*. D, OR = 1.427, 95% CI = 1.229-1.657) and nasopharyngeal carcinoma (II *vs*. DD, OR = 1.339, 95% CI = 1.040-1.724; ID *vs*. DD, OR = 1.257, 95% CI = 1.092-1.447; I *vs*. D, OR = 1.158, 95% CI = 1.002-1.337) in the models. However, we did not find associations in hepatocellular carcinoma, colorectal cancer, bladder cancer, prostate cancer, non-small cell lung cancer and renal cell cancer (Table 2).

**Table 2:** Associations between the NFKB1 −94ins/del ATTG promoter polymorphism and cancer risk

			II+ID *vs*. DD		II *vs*. ID+DD		II *vs*. DD		ID *vs*. DD		I *vs.* D	
Variables	N^a^	Case/Control	OR (95% CI)	I^2^ %	OR (95% CI)	I^2^ %	OR (95% CI)	I^2^ %	OR (95% CI)	I^2^ %	OR (95% CI)	I^2^ %
**Overall**	37	16271/22781	1.139(0.994-1.305)^b^	83.2	1.140(1.029-1.263)b	78	1.259(1.068-1.485)b	84.0	1.118(0.997-1.253)^b^	72.6	1.109(1.025-1.199)b	84.2
**Ethnicity**												
Asian	26	13202/16197	1.223(1.031-1.451)b	87.3	1.280(1.142-1.435)b	76.3	1.463(1.196-1.788)b	86.6	1.151(0.999-1.327)^b^	78.8	1.199(1.092-1.317)b	86.0
Caucasian	11	3069/6584	0.957(0.847-1.081)	27.5	0.824(0.752-0.903)	39.9	0.855(0.748-0.979)	36.2	1.045(0.918-1.188)	24.8	0.899(0.844-0.958)	36.1
**Cancer types**												
Colorectal cancer	5	2777/4409	1.025(0.796-1.319)^b^	68.3	0.890(0.675-1.173)^b^	85	0.947(0.660-1.360)^b^	81.4	1.103(0.959-1.269)	49.9	0.946(0.785-1.140)^b^	84.4
Bladder cancer	4	1788/1955	0.827(0.464-1.475)^b^	90.3	0.983(0.782-1.236)^b^	60.8	0.893(0.510-1.564)^b^	87.1	0.830(0.494-1.394)^b^	86.3	0.948(0.733-1.227)^b^	85.7
Ovarian cancer	4	1463/1573	1.481(1.128-1.943)b	51.6	1.503(1.265-1.786)	0	1.761(1.420-2.184)	39.8	1.246(1.048-1.482)	37.9	1.308(1.181-1.449)	38.5
Prostate cancer	4	2207/2358	1.099(0.753-1.604)^b^	82.0	1.266(0.978-1.639)^b^	57.6	1.382(0.864-2.210)^b^	78.2	1.039(0.797-1.355)^b^	59.1	1.138(0.955-1.357)^b^	69.5
Gastric cancer	2	583/549	0.997(0.260-3.826)^b^	94.3	1.353(0.434-4.221)^b^	91.8	1.275(0.195-8.331)^b^	95.5	0.879(0.295-2.613)^b^	90.4	1.116(0.447-2.784)^b^	95.6
Oral squamous cell carcinoma	2	674/721	1.593(1.253-2.026)	3.9	1.674(1.292-2.169)	0	2.104(1.545-2.867)	33.0	1.420(1.102-1.829)	0	1.427(1.229-1.657)	6.9
None small cell lung cancer	2	516/524	0.779(0.155-3.921)^b^	89.8	1.005(0.497-2.033)^b^	78.6	0.778(0.124-4.904)^b^	91.0	0.806(0.187-3.478)^b^	86.6	0.955(0.453-2.017)^b^	90.5
Hepatocellular carcinoma	3	961/2530	1.503(0.907-2.492)^b^	82.4	1.699(0.873-3.307)^b^	92.2	2.022(0.861-4.746)^b^	91.8	1.179(0.962-1.445)	44.9	1.442(0.916-2.271)^b^	92.8
Nasopharyngeal Carcinoma	3	2659/3088	1.200(0.883-1.631)^b^	73.7	1.146(0.918-1.431)^b^	65.4	1.339(1.040-1.724)b	52.0	1.257(1.092-1.447)	0	1.158(1.002-1.337)b	63.2
Rental cell cancer	2	1356/1895	0.947(0.564-1.591)^b^	65.8	0.991(0.857-1.146)	1.9	0.948(0.764-1.176)	0	1.071(0.644-1.780)^b^	61.8	0.981(0.886-1.086)	0
Other cancers	6	1287/3179	1.174(0.851-1.619)^b^	61.5	0.952(0.704-1.286)^b^	73.1	1.105(0.705-1.733)^b^	75.0	1.218(1.003-1.480)	24.7	1.029(0.822-1.288)^b^	78.2

The bold values indicate that the association is significant

a Number of comparisons

b Random-effect model

### Publication bias

**Figure 4:**
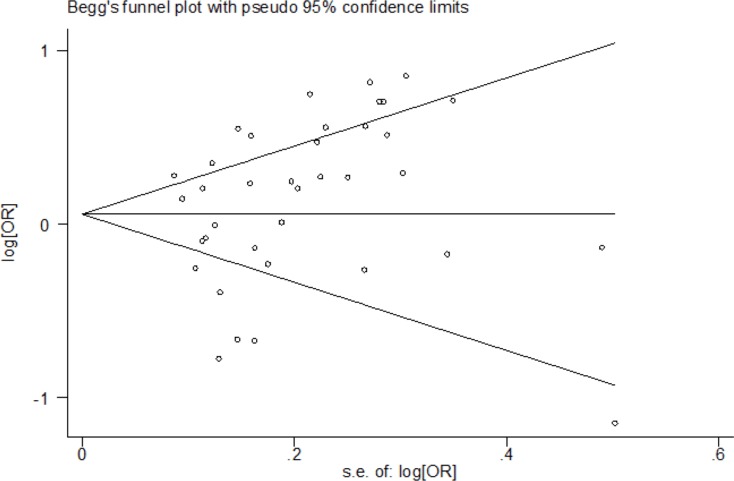
Begg's funnel plot of the association between the NFKB1 −94ins/del ATTG promoter polymorphism and risk of cancer (II + ID *vs*. DD)

The publication bias analysis was performed by Begg's funnel plot and Egger's test. The shape of the Begg's funnel plots seemed symmetrical (Figure 4) and Egger's test suggested no evidence of significant publication bias (*p* = 0.161 for the dominant model, *p* = 0.056 for the recessive model, *p* = 0.092 for the homozygote model, *p* = 0.239 for the heterozygote model, and *p* = 0.117 for the allele model) in this updated meta-analysis.

### Sensitivity analysis

The sensitivity analysis was performed by the sequential omission of individual studies. After excluding each study sequentially, we obtained statistically similar results (data not shown), suggesting that the data of our meta-analysis are relatively stable and credible. In addition, the random-effects model was compared with the fixed-effects model, and the statistically similar results were obtained in all genetic models.

## DISCUSSION

In recent years, several investigators reported the association between the *NFKB1* −94ins/del ATTG promoter polymorphism and risk of cancers [14–35] such as bladder, ovarian, prostate, gastric and breast cancers as well as non-small cell lung, hepatocellular and nasopharyngeal carcinomas, but the results are inconclusive. Previous meta-analyses [45–48] had the drawback of a limited number of studies included and small sample sizes, or studies that were not in HWE were not excluded, which may affect the validity of the conclusions. Many relevant case-control studies were published recently [36–44], including more ethnicities and cancer types. However, the results of these articles were not consistent in previous meta-analyses. To provide a more comprehensive conclusion, we expanded the sample size to more than double through the addition of new studies that were published since the previous meta-analyses.

We performed a meta-analysis of 37 case-control studies from 33 articles (16,271 cases and 22,781 controls) to clarify the relationship between the *NFKB1* −94ins/del ATTG promoter polymorphism and cancer susceptibility. We found that the *NFKB1* −94ins/del ATTG promoter polymorphism was significantly associated with increased risk of cancer; this result was different than a previous meta-analysis [48], which reported that there was no association between the *NFKB1* −94ins/del ATTG promoter polymorphism and cancer risk. The reasons for this difference could be explained as follows: 1) we included 37 case-control studies, *versus* only 11 studies (2,743 cases and 2,195 controls) in the previous meta-analysis, and therefore, the results of this meta-analysis were more credible; and 2) there may be some factors among the study populations that could influence the results, including age, gender, life style, and environment. In addition, when compared with the meta-analysis by Wenyuan Duan [45], although we reached the same conclusion in the terms of overall population, our analysis has some advantages: 1) we excluded articles that do not conform to HWE, whereas the previous meta-analyses did not; and 2) we included 37 studies, whereas previous meta-analyses included just 25 studies, which could lead to a lack of statistical power and reliability. However, we must be careful in explaining the results due to the moderate heterogeneity. To investigate the origin of the heterogeneity, we conducted a stratification analysis based on ethnicity and cancer type. In the subgroup analysis of ethnicity, we found a significant association of the *NFKB1* −94ins/del ATTG promoter polymorphism with increased and decreased cancer risk in Asian and Caucasian populations, respectively. Surprisingly, the results were different from the result shown by a previous meta-analysis [45], which conducted that the *NFKB1* −94ins/del ATTG promoter polymorphism was associated with risk in Asians but not in Caucasians population. The results may be explained by the following: 1) this discrepancy may be because of the limited sample size. The previous meta-analysis included only 9 articles (2047 cases and 2040 controls) in Caucasians, whereas we included 11 articles (3069 cases and 6584 controls); 2) we excluded the studies that do not follow HWE. Therefore, the results of this study are more reliable than the previous meta-analysis; 3) The sensitivity analysis was conducted through two methods in this meta-analysis, and the results were consistent with the previous results, suggesting the results of this study were stable.

Although the mechanism was not clear, we assumed that the mechanism underlying the cancer risk was related to the levels of p50. In recent studies [16,68], it was shown that the probable mechanism of the observed association may be relative to the upregulation of the expression and activity of p50, once p50 is over expressed, it may influence cancer risk. However, cancer is a complex disease influenced by genetic and other non-genetic factors such as environment, lifestyle and habits that might influence the incidence ratio of cancer[64–66]. The *NFKB1* −94ins/del ATTG promoter polymorphism was just one of susceptibility genes, and all these non-genetic factors could influence the expression of the gene. Therefore, the differences in this *NFKB1* polymorphism in Asians and Caucasians may result from different genetic background, environment, lifestyle or other factors.

According to the results of the analysis of the relationship between the *NFKB1* −94ins/del ATTG promoter polymorphism and subtypes of cancer, the *NFKB1* −94ins/del ATTG promoter polymorphism is a risk factor for oral squamous cell carcinoma, ovarian cancer and nasopharyngeal carcinoma. This result suggests that the *NFKB1* gene might have some relevance in these cancers. The inconsistent may be caused by their different micro-environment, because the same genetic factor might have different correlations in different cancer site [67]. Our study has a relatively small number of cases in each cancer type, which might create significant or insignificant associations by chance due to insufficient statistical power. Therefore, further research should enlarge the sample for each cancer type and validate the cancer-specificity effect of this functional polymorphism on cancer susceptibility.

This study has several limitations, like any meta-analysis. First, moderate heterogeneity was detected in some comparisons and may distort the meta-analysis. Second, the non-genetic risk factors such as environment are also important in the incidence ratio of cancer. Unfortunately, there were not enough data for further subgroup analysis; therefore, the results of subgroup analysis may affect the validity of the conclusions. Third, in the subgroup analysis, we found that our analysis was limited to Asian and Caucasian populations, so we do not know whether these conclusions can also be adopted in other populations. This may cause publication bias. Finally, the sample sizes for each type of cancer were relatively small, so further research should enlarge the sample sizes to obtain more accurate conclusions.

Despite these limitations, our study has several strengths. First, all of the studies that we chose agreed with HWE, which may increase the validity of the conclusions. Second, the sample size of our study was more than double that of the previous meta-analysis, significantly increasing the statistical power. Although this updated meta-analysis had the above-mentioned shortcomings, we tried to control them through perfected searching, sifting the good ones from the bad and performing the statistical analyses strictly.

## CONCLUSIONS

We conclude that the *NFKB1* −94ins/del ATTG promoter polymorphism is associated with cancer risk not only in Asian populations, but also in Caucasian populations. Moreover, there might be a significant association with increased susceptibility between the *NFKB1* −94ins/del ATTG promoter polymorphism and ovarian cancer, oral squamous cell carcinoma, and nasopharyngeal carcinoma. Well-designed studies with larger representative sample sizes are necessary to confirm our results.

## MATERIALS AND METHODS

The systematic review and meta-analysis was in accordance with the PRISMA (Preferred Reporting Items for Systematic Reviews and Meta-Analyses) guidelines

### Publication search

A systematic search of the PubMed, Web of Science, Science Direct, Ovid, China National Knowledge Infrastructure (CNKI) and Wan fang Data electronic databases was performed with the following key words: (“polymorphisms” OR “polymorphism” OR “SNP” OR “single nucleotide polymorphism” OR “variant” OR “mutation”) AND (“neoplasm” OR “cancer” OR “tumor” OR “carcinoma” OR “carcinogenesis”) AND (“NF-κB1” OR “Nuclear factor-κB1” OR “Nuclear factor κB1” OR “NFKB1” OR “nuclear factor kappa B1” OR “NF kappa B1” OR “nuclear factor kB1” OR “rs28362491”).

### Inclusion criteria

No language or other restrictions were imposed in this study and the inclusion criteria were as follows: 1) case-control design; 2) studies evaluating the association between the *NFKB1* −94ins/del ATTG promoter polymorphism and cancer risk; 3) studies describing the genotype distributions in detail to calculate the OR and 95%CI in cases and controls; and 4) the distribution data in controls must be consistent with Hardy-Weinberg Equilibrium (HWE).

### Exclusion criteria

The exclusion criteria in this meta-analysis were as follows: 1) not concerned with cancer risk; 2) only a case population; 3) duplication of a previous publication; 4) the control group does not conform to HWE; and 5) animal studies.

### Data extraction

According to the criteria listed above, information was carefully extracted from eligible studies independently by each investigator (Y.Q.L. and D.W.). The following information was collected from each study: surname of the first author, year of publication, ethnicity of subjects, genotyping method, frequencies of the genotypes in cases and controls, cancer type. The different ethnicities were categorized as Caucasian or Asian. Studies that investigated more than one type of cancer were regarded as individual datasets only in subgroup analyses according to cancer type. Any discrepancy was resolved through discussion.

### Statistical analysis

The strength of association between the *NFKB1* −94ins/del ATTG promoter polymorphism and cancer was estimated through OR with 95% CI. The combined ORs were determined by the Z test, and a *P* value of <0.05 was considered to be statistically significant. The *NFKB1* −94ins/del ATTG promoter polymorphism consists of three genotypes: homozygote insertion or wild-type (II), homozygote deletion or variant (DD), and heterozygous ins/del (ID). We measured the association based on five different genetic models: the dominant (II+ID *vs*. DD), recessive (II *vs*. ID + DD), homozygote (II *vs*. DD), heterozygote (ID *vs*. DD), and allele (I *vs*. D) models. To investigate the origin of heterogeneity, subgroup analyses based on ethnicity (Caucasian and Asian) and cancer type were performed to identify the association between the *NFKB1* −94ins/del ATTG promoter polymorphism and cancer susceptibility.

We used the *Q* and *I^2^* statistical tests to check the statistical heterogeneity among studies. If the *P* value was < 0.05 and *I^2^* ≥ 50% indicating heterogeneity, then a random-effect model was chosen to calculate the pooled OR; otherwise, a fixed-effect model was selected [12]. A sensitivity analysis was conducted by sequentially excluding each study to evaluate the stability of the results. The publication bias was estimated by Egger's test and Begg's funnel plots, with potential publication bias if *p*<0.05 and the plot was asymmetrical [13]. The statistical analyses were performed using STATA 11.0 software (Stata Corp, College Station, TX, USA).
